# Characterization of the complete chloroplast genome of the invasive plant *Erigeron annuus* (L.) Pers. (Asterales: Asteraceae)

**DOI:** 10.1080/23802359.2021.2018946

**Published:** 2022-01-11

**Authors:** Jianyun Zhou, Juanjuan Li, Shaobing Peng, Xiangming An

**Affiliations:** a College of Forestry, Northwest A&F University, Yangling, People’s Republic of China; b Yangling Vocational and Technical College, Yangling, People’s Republic of China; c Qiaoshan State-owned Forestry Administration, People’s Republic of China

**Keywords:** *Erigeron annuus* (L.) Pers, exotic plant high-throughput sequencing (HTS), invasive plant chloroplast genome

## Abstract

*Erigeron annuus* (L.) Pers. (annual, daisy or tall fleabane) is an annual herb native to North America but has been introduced and naturalized worldwide. In this study, its complete chloroplast (cp) genome was assembled from Illumina sequencing reads. The cp genome is 153,177 bp long with an A + T-biased base composition. It encodes a panel of 113 genes, including 80 protein-coding, 29 tRNA, and four rRNA genes. Nineteen genes are completely or partially duplicated, while 17 genes possess one or two introns. Phylogenetic analysis suggested *E. annuus* is mostly closely related to *Erigeron canadensis* L. and that the two genera *Conyza* and *Erigeron* are not mutually monophyletic.

*Erigeron annuus* (L.) Pers., commonly known as annual, daisy or tall fleabane, is an annual herb species belonging to the family Asteraceae of the order Asterales (Chen et al. 2011). It is native to North America, but has been introduced and naturalized worldwide (Wu et al. 2004; Chen et al. 2011; Vuković 2015; Zimmermann et al. 2015; Seipel et al. 2016; Das et al. 2017; Shhagapsoev et al. 2018; Song et al. 2018; Sennikov and Kurtto 2019). To date, most studies of *E. annuus* have been focused upon its histochemistry (Kim et al. 2005; Yoo et al. 2008; Jeong et al. 2011; Kim and Choi 2015; Kim et al. 2018), invasive biology (Song et al. 2018; Sennikov and Kurtto 2019; Wei et al. 2020) and population genetics (Stratton 1991, 1992; Edwards et al. 2006; Tunaitienė et al. 2017). Little is known about its genomics (incl. chloroplast/cp genomics). In this study, we assembled the first complete cp genome for this invasive weed using high-throughput sequencing technology, and investigated its phylogenetic placement within the tribe Astereae (Asterales: Asteraceae).

Fresh leaves were sampled from an individual of *E. annuus* in Xunyangba Village, Ningshan County, Shaanxi Province, China (33°32'38′'N, 108°32'22′'E), and were used to isolate the total genomic DNA with the DNeasy Plant Mini Kit (Qiagen, CA, USA). A voucher specimen was held at herbarium of the College of Forestry, Northwest A&F University (https://en.nwsuaf.edu.cn/; Juanjuan Li, Email: wutong761014@163.com) under the accession number EANNU-2019-07-22. Library construction and Illumina PE150 sequencing (average insert size: 350 bp) were performed by Beijing Novogene Technology Co., Ltd. (Beijing, China) following the protocol of the manufacturer (Illumina, CA, USA). In all, 22.13 M of paired-end reads were retrieved, and were used to assemble the cp genome of *E. annuus* with the software NOVOPlasty v4.3.1 (Dierckxsens et al. 2017). The cp genome of *Sonchus webbii* Sch. Bip. (GenBank accession: MK033508) (Cho et al. 2019) was inputted as the initial seed sequence. Annotation of the cp genome was done in Geneious R11 (Biomatters Ltd., Auckland, New Zealand) by aligning with those of closely related taxa.

The cp genome of *E. annuus* is 153,177 bp in length, and comprises a pair of inverted repeat (IR) regions (25,019 bp) which were separated by a large single-copy (LSC) region (84,727 bp) and a small single-copy (SSC) region (18,412 bp). The base composition is asymmetric with the A + T contents of the entire genome, the SSC, LSC and IR regions being 63.0%, 69.1%, 65.1% and 57.0%, respectively. The cp genome encodes a panel of 113 genes [incl. 80 protein-coding (PCG), 29 tRNA and four rRNA genes]. In all, 17 genes are completely duplicated, including seven PCGs (*ndh*B, *rpl*2, *rpl*23, *rps*7, *rps*12, *ycf*2, and *ycf*15), six tRNAs (*trn*A-UGC, *trn*E-UUC, *trn*I-CAU, *trn*L-CAA, *trn*R-ACG, and *trn*V-GAC) and all four rRNAs (*rrn*4.5, *rrn*5, *rrn*16 and *rrn*23). Two PCGs (*rps*19 and *ycf*1) are partially duplicated. In addition, a single intron is presented in ten PCGs (*atp*F, *ndh*A, *ndh*B, *pet*B, *pet*D, *rpl*2, *rpl*16, *rpo*C1, *rps*12, and *rps*16) and five tRNA genes (*trn*A-UGC, *trn*E-UUC, *trn*G-ACC, *trn*K-UUU, and *trn*L-UAA), and a couple of introns are presented in two PCGs (*clp*P and *ycf*3).

Phylogenetic analyses were conducted based on the coding sequences of cp PCGs to ascertain the phylogenetic placement of *E. annuus* within the tribe Astereae (Figure 1). Both Bayesian inference (BI) and maximum-likelihood (ML) methods were implemented using the software MrBayes v3.1.1 (Ronquist and Huelsenbeck 2003) as in TOPALi v2.5 (Milne et al. 2009) and the software MEGA11 (Tamura et al. 2021). The key parameters for BI analysis were set as follows: <Nucleotide substitution model: GTR±G ± I; Runs: 4; Generations: 200,000; Sample Freq.: 10; Burnin: 30%>, and those for ML analysis were set as follows: < Nucleotide substitution model: GTR±G ± I; Number of bootstrap replications: 500>. The nucleotide substitution models for BI and ML methods were inferred with the ‘Model Selection (MrBayes)’ function in TOPALi v2.5 and the ‘Find Best DNA/Protein Models (ML)’ function in MEGA11, respectively. The outgroup taxa used in this study are four species within the tribe Anthemideae (Asterales: Asteraceae), including *Artemisia capillaris* Thunb. (KU736963), *Chrysanthemum boreale* Makino (MG913594), *Ismelia carinata* (Schousb.) Sch. Bip. (MG710387) and *Soliva sessilis* Ruiz & Pav. (KX063863). The BI and ML analyses recovered the identical topology. *E. annuus* was found to be mostly closely related to the congener *E. canadensis*. In addition, the two genera *Conyza* and *Erigeron* were not mutually monophyletic but together formed a monophyletic clade. This finding appears to support the inclusion of *Conyza* within the genus *Erigeron* (Noyes 2000). However, further studies based on more extensive sampling are necessary to resolve this controversy.

**Figure 1.**
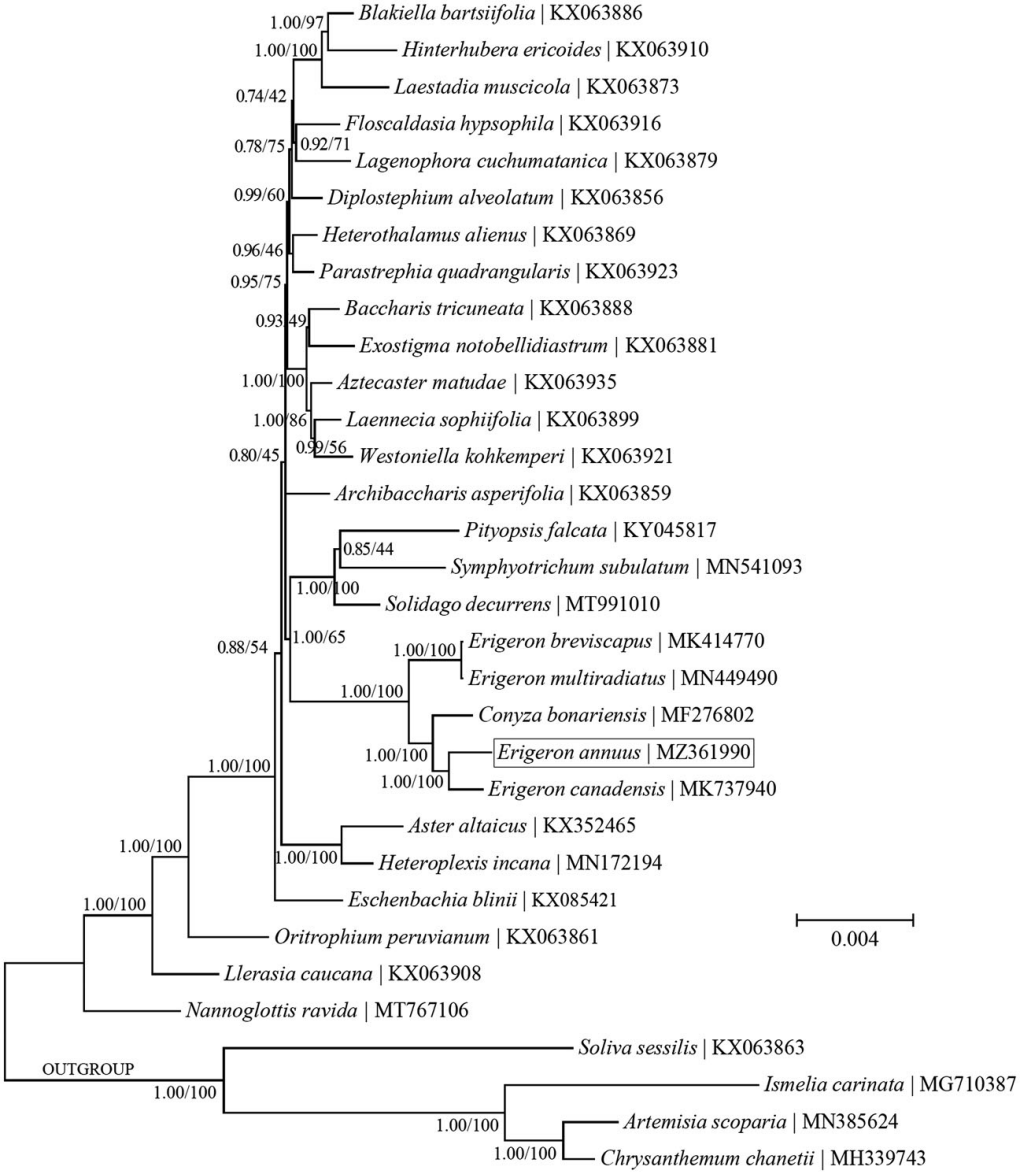
A combined phylogeny of the tribe Astereae based on the Bayesian inference (BI) and maximum-likelihood (ML) analysis of chloroplast protein-coding genes. The BI and ML analyses recovered the identical topology. The support values next to the nodes were Bayesian posterior probabilities according to the BI analysis (first value) and bootstrap percentages of 500 pseudoreplicates according to the ML analysis (second value). Four species within the tribe Anthemideae were included as the outgroup taxa.

## Data Availability

The genome sequence data that support the findings of this study are openly available in GenBank of NCBI at [https://www.ncbi.nlm.nih.gov] under the accession number MZ361990. The associated BioProject, SRA and Bio-Sample numbers are PRJNA736110, SRR14763427 and SAMN19609949, respectively.
